# Baclofen Promotes Osteochondrogenic Commitment of Mesenchymal Stem Cells: Implications for Heterotopic Ossification Risk

**DOI:** 10.3390/ijms27062783

**Published:** 2026-03-19

**Authors:** María Crugeiras-Sampedro, Lorena Zas-Veiga, María Piñeiro-Ramil, Andrés Pazos-Pérez, Verónica López-López, Alberto Jorge-Mora, Ana Alonso-Pérez, Rodolfo Gómez

**Affiliations:** 1 Musculoskeletal Pathology Group, Health Research Institute of Santiago de Compostela (IDIS), Santiago University Clinical Hospital, Servizo Galego de Saude (SERGAS), 15706 Santiago de Compostela, Spain; maria.sampedro.sampedro@rai.usc.es (M.C.-S.); lorena.zas@rai.usc.es (L.Z.-V.); maria.ramil@idisantiago.es (M.P.-R.); andres.pazos.perez@sergas.es (A.P.-P.); veronica.lopez.lopez1@sergas.es (V.L.-L.); alberto.agustin.jorge.mora@sergas.es (A.J.-M.); 2 Traumatology and Orthopedics Service, A Coruña University Clinical Hospital, Servizo Galego de Saude (SERGAS), 15006 A Coruña, Spain

**Keywords:** heterotopic ossification, spasticity, baclofen, tizanidine, mesenchymal stem cells, adipogenesis, osteochondrogenesis, inflammation, ectopic bone formation and MSC differentiation

## Abstract

(1) Heterotopic ossification (HO) is a pathological process characterized by ectopic bone formation in soft tissues, often following trauma or neurological injury, and is associated with spasticity and chronic inflammation. Mesenchymal stem cells (MSCs) play a central role in HO by differentiating into osteoblasts through endochondral or intramembranous ossification, while alternative fates such as adipogenesis are suppressed. In this study, we investigated the effects of two commonly used antispastic drugs, baclofen and tizanidine, on MSC differentiation under adipogenic and inflammatory conditions in vitro. (2) Mouse C3H10T1/2 MSCs were cultured and induced toward adipogenesis in the presence of baclofen or tizanidine, and inflammatory stimuli (Interleukin-1β or lipopolysaccharides) were applied where indicated. Gene expressions of adipogenic and osteochondrogenic markers were assessed by RT-qPCR, while osteopontin protein levels were quantified by Simple Western. (3) Baclofen treatment significantly inhibited adipogenic gene expression and promoted osteochondrogenic markers and osteopontin protein under basal conditions, whereas tizanidine had minimal effects. Under inflammatory conditions, baclofen partially suppressed adipogenesis but did not strongly induce osteochondrogenesis. (4) These findings indicate that baclofen can directly modulate MSC fate, potentially contributing to HO risk, while tizanidine may offer a safer alternative for spasticity management in patients at risk of ectopic bone formation.

## 1. Introduction

Heterotopic Ossification (HO) is a pathological process characterized by the formation of mature lamellar bone in non-skeletal tissues, such as muscles and other soft tissues, mostly around large joints [1,2,3]. HO is an aberrant tissue repair in which normal regeneration is dysregulated, triggering the formation of ectopic bone. This pathology can have a genetic origin, as observed in rare disorders such as fibrodysplasia ossificans progressiva (FOP) and progressive osseous heteroplasia (POH), but the most prevalent forms are acquired and commonly result from severe trauma, particularly neurological injuries [4,5]. The most common affected regions are the hips and the knees (about 60–70% and 20–30% respectively), but it can also appear in elbows and shoulders [1]. HO constitutes a highly debilitating condition, as ectopic bone formation is frequently associated with periarticular inflammation, chronic pain, progressive loss of joint mobility with a possibility of evolving into ankylosis [6]. Because of these, patients develop severe functional limitations that substantially compromise their quality of life and rehabilitation outcomes.

Ectopic bone formation in HO mimics normal physiological skeletal development through endochondral and/or intramembranous ossification in an aberrant anatomical context [7]. Endochondral ossification involves a transient cartilaginous template in which mesenchymal cells (MSCs) differentiate into chondrocytes that progressively mature toward hypertrophic chondrocytes, ultimately undergoing matrix mineralization. After that, osteoblasts are recruited to replace the cartilaginous scaffold by bone [8]. On the contrary, intramembranous ossification occurs through the direct differentiation of MSCs into osteoblasts [9,10,11].

These mechanisms are characterized by the expression of osteochondrogenic genes such as Collagen type II alpha 1 (*COL2A1*), SRY-box transcription factor 9 (*SOX9*), Collagen type X (*COLX*), Bone gamma-carboxyglutamate protein (*BGLAP*), Parathyroid hormone 1 receptor (*PTHR*) and Osteopontin (SPP1) [12,13,14,15,16] Specifically, SPP1 has been described as a biomarker of heterotopic lesions, potentially contributing to disease progression [15,16]. Because MSCs retain the capacity to adopt alternative lineages, including adipogenesis, alterations in MSC fate decisions may modulate the relative contribution of osteogenic versus non-osteogenic programs during ectopic bone formation [2].

Several studies have associated HO with three essential conditions: the presence of osteogenic precursor cells, an inducing osteogenic stimulus and a permissive environment [17]. Within this context, the differentiation potential of MSCs plays a critical role in ectopic bone formation [10,11]. Under physiological conditions, osteogenic and adipogenic differentiations are tightly interconnected and mutually exclusive, so commitment to one lineage occurs at the expense of the other [10,11,18,19,20]. In HO, cell differentiation is strongly shifted towards osteoblastogenesis, and alternative fates are heavily constrained [2,21].

Certain degree of inflammation also plays a critical role in the development and progression of the disease [2,22]. Nonetheless, chronic or excessive inflammation has a significant negative impact on bone growth and remodeling [23]. Inflammatory stimuli, including lipopolysaccharides (LPS) and interleukin-1β (IL-1β), have been implicated in these effects [23,24,25]. They strongly inhibit osteochondrogenesis while promoting other opposite differentiation process like adipogenesis, disrupting the normal balance of bone homeostasis [26,27,28].

In the context of neurological injuries, several factors such as altered tissue perfusion and the release of neuropeptides create a permissive microenvironment for aberrant osteogenesis [2,29]. In patients with alterations in neural regulation, changes in muscle tone play an important role [3,30]. Spasticity, a common consequence of central nervous system (CNS) injuries, has been frequently linked with the development of HO in patients [3,30,31].

Spasticity is a motor disorder and a stretch reflex abnormality, considered one of the main components of Upper Motor Neuron Syndrome [32,33]. Traditionally, spasticity is characterized by a velocity-dependent increase in tonic stretch reflexes, accompanied by exaggerated tendon reflexes [32,33,34,35]. Clinically, spasticity presents as increased resistance to passive movement that intensifies with speed and is often accompanied by hyperreflexia and clonus [34,36,37]. Beyond its direct clinical significance, spasticity is recognized as a well-established risk factor for the development of HO [3,38,39]. Clinical studies in patients who have suffered spinal cord injury (SCI) have shown that spasticity is independently associated with HO [31].

Pharmacological treatments for spasticity include systemic and local therapies [31,32,33,38,40]. Within the range of pharmacological options, baclofen remains one of the most used agents. It acts as a GABA-B (γ-aminobutyric acid type B) receptor agonist on pre- and postsynaptic neurons in both the central and peripheral nervous systems, inhibiting mono- and polysynaptic reflex transmission at the spinal level [32,40]. On the other hand, tizanidine is a widely used drug that is considered a first-line treatment along with Baclofen. It acts as a central α2-adrenergic receptor agonist, exerting its effects at both spinal and supraspinal levels [34,35]. In patients with traumatic brain injury, large cohort studies have shown that the use of antispastic medications is associated with a higher incidence of HO, highlighting abnormal muscle activity as a contributing factor in ectopic bone formation [38].

As previously noted, spasticity is a recognized risk factor for HO. At the same time, antispastic medications, while used to manage spasticity, have also been described as potential risk factors for HO [32]. Differentiating the contributions of the underlying neurological pathology from the effects of pharmacological treatment is essential. In this context, in vitro studies enable the isolation of these factors and the exploration of how pharmacological interventions influence the underlying cellular and molecular mechanisms. In this work, our main objective was to isolate the effects of antispastic treatments on HO by evaluating their impact on osteochondrogenic differentiation. Here, we show that the antispastic baclofen promotes osteochondrogenic differentiation, even under hostile conditions, such as adipogenic environments—the most antithetical fate to osteoblastogenesis [8,19,21]—whereas under inflammatory conditions, the effect is only partial and fails to clearly induce osteochondrogenesis, while adipogenesis is inhibited in both contexts. In contrast, tizanidine, another commonly used antispastic agent, showed no relevant effect on either the adipogenesis differentiation process or the osteochondrogenesis process.

## 2. Results

### 2.1. Baclofen Treatment Diminishes Adipogenesis and Promotes Osteochondrogenic Gene Expression

Baclofen is an antispastic drug commonly used in patients after big trauma, and tizanidine is considered the only other first-line oral drug for the treatment of spasticity. Antispastics have been described as a risk factor for HO development [32], but it is not possible to separate their impact on the HO from the effect of the spasticity. Thus, we studied the effect of baclofen and tizanidine on adipogenesis to investigate whether these drugs can reprogram MSCs from adipogenesis, the cell fate most antithetic to osteoblastogenesis, a key process in ossification [21].

**Figure 1:**
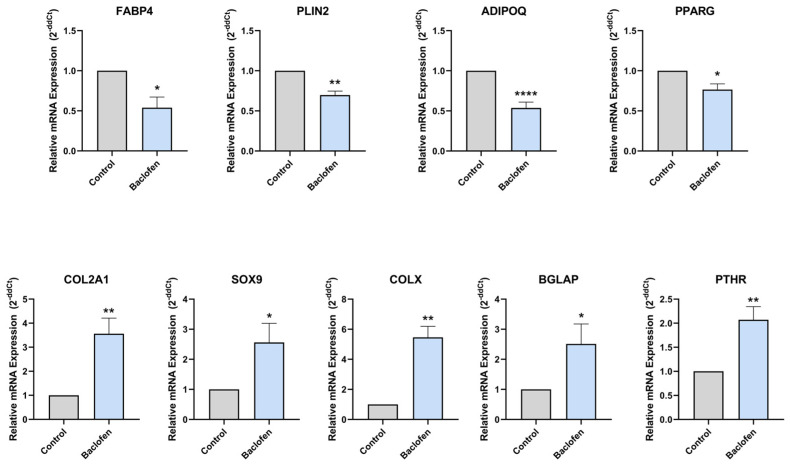
Baclofen effect on adipogenesis. Relative expression of mRNA of Fatty Acid Binding Protein 4 (*FABP4*), Perilipin-2 (*PLIN2*), Adiponectin (*ADIPOQ*), Peroxisome Proliferator-Activated Receptor Gamma (*PPARG*), Collagen type II alpha 1 (*COL2A1*), SRY-box transcription factor 9 (*SOX9*), Collagen type X (*COLX*), Bone gamma-carboxyglutamate protein (*BGLAP*) and Parathyroid hormone 1 receptor (*PTHR*) in C3H10T1/2 cell line differentiated to adipocyte and treated with Baclofen (100 μM) during 7 days. Gene expression levels were normalized to untreated control. Data are presented as mean ± SEM of n = 3 independent experiments. Statistical significance was assessed by two-tailed Student’s *t*-test comparing treated samples to untreated control: (*) *p* < 0.05, (**) *p* < 0.01, (****) *p* < 0.0001.

**Figure 2:**
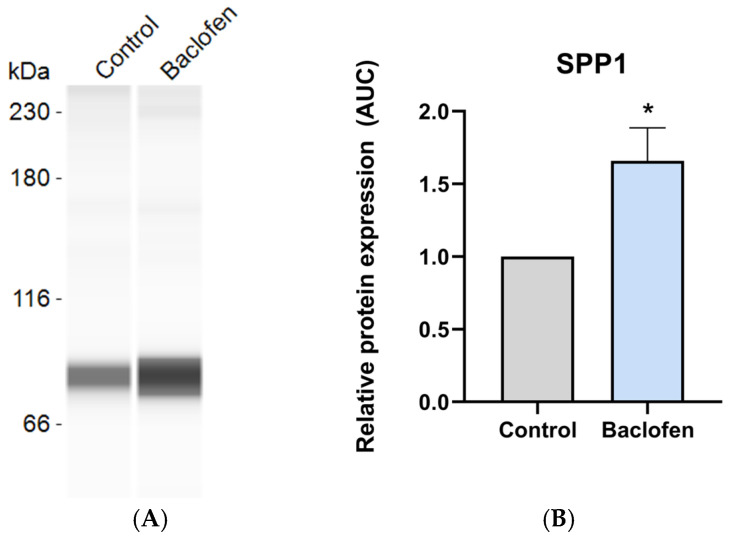
Baclofen effect on SPP1 protein expression. (**A**) Protein expression of Osteopontin (SPP1) in the supernatant of C3H10T1/2 cell line differentiated to adipocyte and treated with Baclofen (100 μM) during 7 days; (**B**) Relative protein expression of SPP1. Gene expression levels were normalized to untreated control. Data are presented as mean ± SEM of n = 3 independent experiments. Statistical significance was assessed by two-tailed Student’s *t*-test comparing treated samples to untreated control: (*) *p* < 0.05.

**Figure 3:**
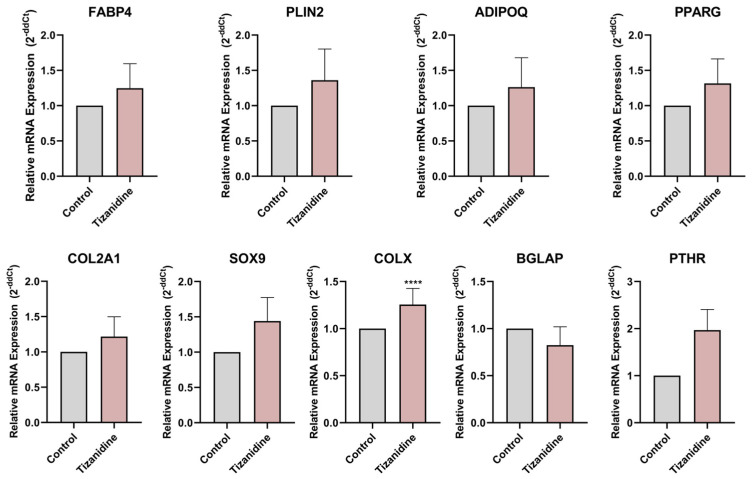
Tizanidine effect on adipogenesis. Relative expression of mRNA of Fatty Acid Binding Protein 4 (*FABP4*), Perilipin-2 (*PLIN2*), Adiponectin (*ADIPOQ*), Peroxisome Proliferator-Activated Receptor Gamma (*PPARG*), Collagen type II alpha 1 (*COL2A1*), SRY-box transcription factor 9 (*SOX9*), Collagen type X (*COLX*), Bone gamma-carboxyglutamate protein (*BGLAP*) and Parathyroid hormone 1 receptor (*PTHR*) in C3H10T1/2 cell line differentiated to adipocyte and treated with Tizanidine (10 μM) during 7 days. Gene expression levels were normalized to untreated control. Data are presented as mean ± SEM of n = 3 independent experiments. Statistical significance was assessed by two-tailed Student’s *t*-test comparing treated samples to untreated control: (****) *p* < 0.0001.

**Figure 4:**
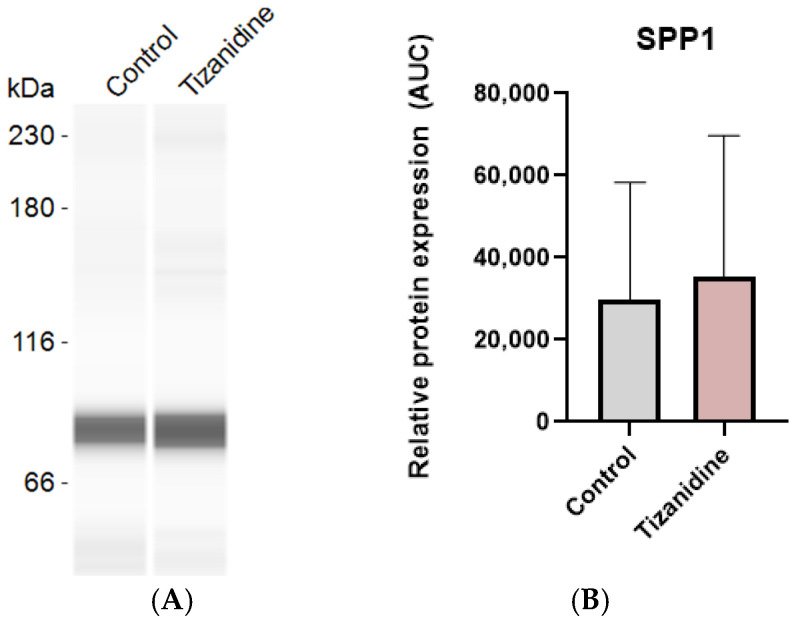
Tizanidine effect on SPP1 protein expression. (**A**) Protein expression of Osteopontin (SPP1) in the supernatant of C3H10T1/2 cell line differentiated to adipocyte and treated with Tizanidine (10 μM) during 7 days; (**B**) Relative protein expression of SPP1. Data are expressed as mean ± SEM of n = 3 independent experiments.

After 7 days of differentiating C3H10T1/2 MSC into adipocytes in the presence or absence of baclofen or tizanidine, mRNA expression levels of *FABP4*, *PLIN2*, *ADIPOQ*, *PPARG*, *COL2A1*, *SOX9*, *COLX*, *BGLAP*, and *PTHR* were measured by RT-qPCR. As shown in Figure 1, MSC differentiation in the presence of baclofen (100 µM) significantly reduced the expression of *FABP4*, *PLIN2*, *ADIPOQ*, and *PPARG* adipogenic marker genes, while significantly increased the expression of osteochondrogenic marker genes: *COL2A1*, *SOX9*, *COLX*, *BGLAP*, and *PTHR*. Consistent with these results, we determined the effect of baclofen on the production of SPP1 into the cell supernatant. Data revealed that baclofen significantly increased SPP1 protein expression when compared with untreated cells (Figure 2). In contrast, treatment with tizanidine (10 μM) did not modulate the expression of the studied adipogenic genes and only induced a mild increase in the expression of *COLX* gene when compared with the untreated control (Figure 3). Finally, as shown in Figure 4, tizanidine treatment did not lead to any significant changes in the expression of SPP1 protein compared with untreated control cells.

### 2.2. Baclofen Does Not Clearly Affect Osteochondrogenesis Under Inflammation

Although inflammation is present after trauma and has been described as a risk factor for ossification and HO development, excessive inflammation has been associated with the inhibition of bone formation [3,17]. Therefore, considering the strong effects of baclofen we decided to evaluate these effects in the context of a strong and sustained inflammatory environment, which is well-known disruptor of osteochondrogenic processes [8].

C3H10T1/2 MSCs were differentiated into adipocytes for 7 days in the presence or absence of baclofen, and two strong inflammatory stimuli (IL-1β (0.5 ng/mL) or LPS (100 ng/mL)). mRNA expression levels of adipogenic (*FABP4*, *PLIN2*, *ADIPOQ*, *PPARG*) and osteochondrogenic (*COL2A1*, *SOX9*, *COLX*, *BGLAP* and *PTHR*) marker genes were measured by RT-qPCR.

**Figure 5:**
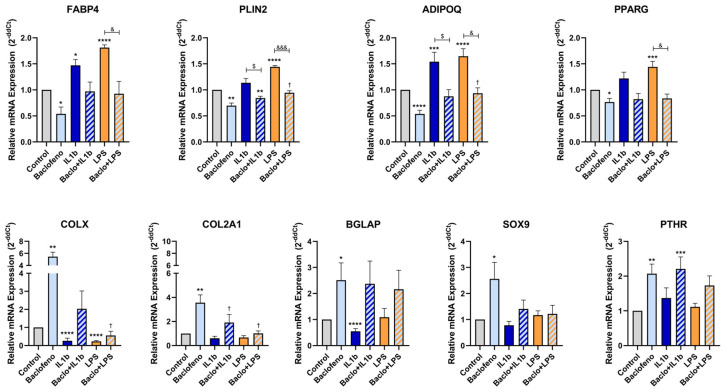
Baclofen modulates inflammation effects on adipogenesis genetic profile. Relative expression of mRNA of Fatty Acid Binding Protein 4 (*FABP4*), Perilipin-2 (*PLIN2*), Adiponectin (*ADIPOQ*), Peroxisome Proliferator-Activated Receptor Gamma (*PPARG*), Collagen type II alpha 1 (*COL2A1*), SRY-box transcription factor 9 (*SOX9*), Collagen type X (*COLX*), Bone gamma-carboxyglutamate protein (*BGLAP*) and Parathyroid hormone 1 receptor (*PTHR*) in 7 days C3H10T1/2 adipogenesis treated with Interleukin-1β (0.5 ng/mL), baclofen (100 μM), Interleukin-1β with baclofen, LPS (100 ng/mL) and LPS with baclofen (n = 3). Gene expression levels were normalized to untreated control. Data are presented as mean ± SEM of n = 3 independent experiments. Statistical significance was assessed by two-tailed Student’s *t*-test: (*) *p* < 0.05, (**) *p* < 0.01, (***) *p* < 0.001 and (****) *p* < 0.0001 regarding untreated control; ($) *p* < 0.05 regarding Interleukin-1β; (&) *p* < 0.05 and (&&&) *p* < 0.001 regarding LPS; (†) *p* < 0.05 regarding baclofen.

As depicted in Figure 5, treatment with IL-1β significantly enhanced the expression of *FABP4* and *ADIPOQ* adipogenic marker genes and significantly reduced the expression of *COLX* and *BGLAP* osteochondrogenic genes, compared to the untreated control. In contrast, co-treatment with baclofen and IL-1β led to a significant reduction in the expression of *PLIN2* and *ADIPOQ* adipogenic gene relative to cells treated with IL-1β alone.

Studying TLR4-mediated inflammation (Figure 5), treatment with LPS resulted in a significant increase on the expression of adipogenic genes *FABP4*, *PLIN2*, *ADIPOQ* and *PPARG*, while the expression of *COLX* osteochondrogenic gene significantly decreased compared to the untreated control. Finally, co-treatment with baclofen and LPS significantly decreased the expression of *FABP4*, *PLIN2*, *ADIPOQ* and *PPARG*, demonstrating significant differences compared with the LPS-treated group.

**Figure 6:**
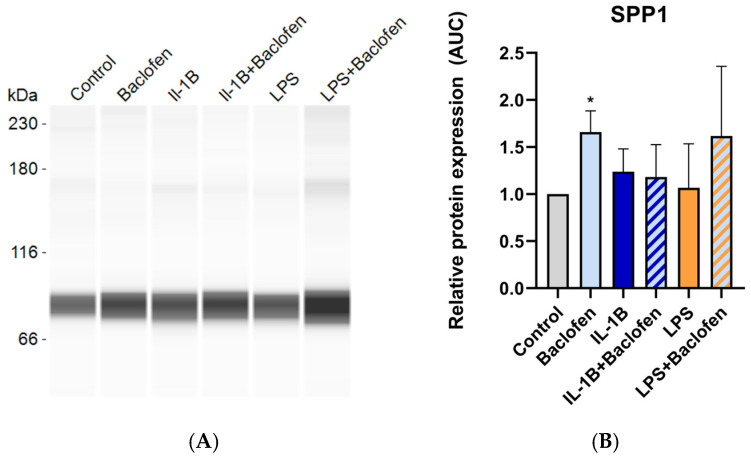
Inflammation effects on SPP1 protein expression. (**A**) Protein expression of Osteopontin (SPP1) in the supernatant of C3H10T1/2 cell line differentiated to adipocyte and treated with Interleukin-1β (0.5 ng/mL), baclofen (100 μM), Interleukin-1β with baclofen, LPS (100 ng/mL) and LPS with baclofen (n = 3); (**B**) Relative protein expression of SPP1. Gene expression levels were normalized to untreated control. Data are presented as mean ± SEM of n = 3 independent experiments. Statistical significance was assessed by two-tailed Student’s *t*-test comparing treated samples to the untreated control: (*) *p* < 0.05.

**Figure 7:**
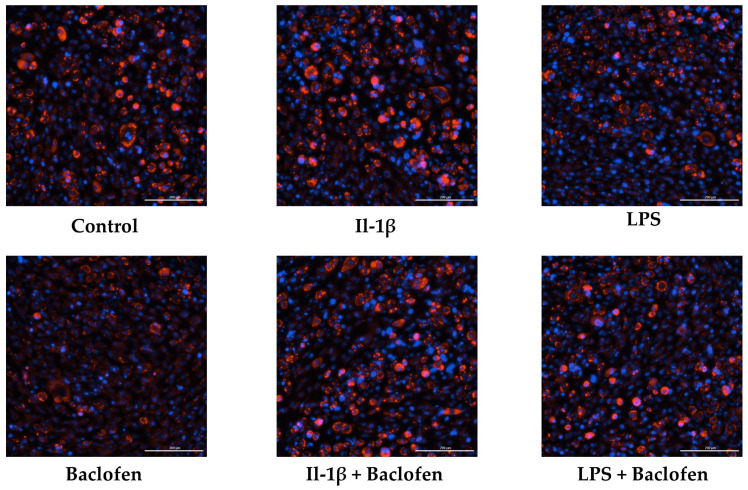
Lipid droplet accumulation under inflammatory conditions. Representative fluorescence microscopy images of the C3H10T1/2 cell line treated with interleukin-1β (0.5 ng/mL), baclofen (100 μM), interleukin-1β with baclofen, LPS (100 ng/mL) and LPS with baclofen.

Finally, Figure 6 shows that baclofen alone significantly increased SPP1 protein expression, whereas treatment with IL-1β, LPS or their combination with baclofen did not affect SPP1 levels compared with untreated controls. These findings were further supported by fluorescence microscopy analysis (Figure 7), which revealed a consistent pattern of lipid droplet accumulation, with differences observed only in the presence of baclofen.

## 3. Discussion

Here we show that the antispastic baclofen affects the differentiation of MSCs to adipocytes by inhibiting adipogenic marker gene expression while promoting the expression of osteochondrogenic marker genes. In these cells, baclofen also increased the secretion of SPP1, a well-known protein associated with heterotopic ossification [15]. Notably, the anti-adipogenic effect of baclofen was preserved even in the context of a sustained and robust inflammatory environment (with IL-1β or LPS). In contrast, tizanidine, another commonly used antispastic agent, showed no relevant effect on either the adipogenesis differentiation process or the osteochondrogenesis process.

Heterotopic ossification is a highly debilitating condition characterized by the formation of mature bone in periarticular soft tissues [3,22], leading to chronic inflammation, persistent pain and progressive loss of joint mobility, which significantly compromises quality of life and rehabilitation outcomes, particularly in patients with neurological injuries [22,23].

HO has been reported in patients with spasticity, particularly in patients with spinal cord injury or traumatic brain injury, where abnormal muscle activity creates a mechanical and inflammatory environment that promotes ectopic bone formation [3,23]. However, in clinical practice, these patients are usually treated with antispastic drugs, so it is not possible to determine the respective contributions of each factor to the development of HO, or whether a synergistic effect exists between them [32].

For this reason, an experimental approach that allows independent analysis of the effects of antispastic drugs is necessary. In this study, we employed in vitro models, taking advantage of the physiological balance of MSCs between osteoblastogenesis and adipogenesis, and used an adipogenic context—opposed to osteoblastogenesis—to assess whether these drugs can interfere with MSC differentiation, either by promoting the activation of aberrant osteochondrogenic programs involved in ectopic bone formation, or by inhibiting other differentiation pathways [20,21]. Importantly, ectopic bone formation occurs in environments that are considerably less favorable than those supporting physiological skeletal bone formation; therefore, we explored the ability of antispastic drugs to promote osteochondrogenic programs under particularly hostile conditions, such as adipogenic differentiation in the presence of sustained inflammation.

Baclofen is a γ-aminobutyric acid (GABA) derivative that acts as a selective agonist of GABA-B receptor [41]. Although these receptors are best known for their role in the nervous system, increasing evidence indicates that GABA-B signaling is also involved in the regulation of skeletal tissues [42,43,44]. GABA-B receptors have been shown to play a stimulatory role in cartilage growth and the activation of functional receptors by agonists such as baclofen enhances osteochondrogenic cell proliferation [44,45], which is consistent with the activities of baclofen in our in vitro model.

Treatment of C3H10T1/2 MSCs under adipogenic conditions with baclofen led to a clear reduction in the expression of adipogenic marker genes, including *FABP4*, *PLIN2*, *ADIPOQ* and *PPARG*, together with an increase in genes associated with osteochondrogenic differentiation such as *COL2A1*, *SOX9*, *COLX*, *BGLAP* and *PTHR*. Overall, these results indicate that baclofen alters the differentiation of MSCs, shifting it away from adipogenesis and toward an osteochondrogenic program. Consistent with this, fluorescence analysis showed a decrease in lipid droplet accumulation. To our knowledge, this is the first evidence showing that baclofen modulates adipogenic differentiation.

At protein level, baclofen significantly increased SPP1 secretion. Given that SPP1 is involved in cell–matrix interactions and mineralization during ectopic bone formation [15,46], its increased expression supports that baclofen can influence MSC behavior by promoting the secretion of factors that might contribute to the establishment of a permissive environment for ectopic bone formation.

The osteochondrogenic markers selected for this study included *SOX9* and *COL2A1*, indicative of chondrogenesis [9,47]; *COLX*, a marker of hypertrophic chondrocytes and endochondral ossification [13]; *BGLAP* and SPP1, associated with osteoblast differentiation [15,46]; and *PTHR*, involved in the regulation of bone metabolism [48].

Thus, baclofen increased expression of *SOX9*, *COL2A1* and *COLX*, suggests that baclofen truly promotes commitment toward the osteochondrogenic lineage [8,9,23]. In addition, the upregulation of *BGLAP* and *PTHR* points to a possible involvement of GABA-B signaling in osteoblast differentiation and bone formation [49]. Although the role of GABA-B receptors in osteoblasts appears to be more complex and remains partially controversial, available evidence supports a positive contribution of GABA signaling to osteogenesis, with receptor activation being required for proper mineralization [43].

Since adipogenic and osteochondrogenic differentiation represent competing outcomes in MSCs, the activation of an osteochondrogenic program is likely to contribute to the observed suppression of adipogenic markers [19]. Taken together, these findings suggest that baclofen influences MSCs fate decisions by reducing adipogenic differentiation and supporting osteochondrogenic outcomes that could be mediated through GABA signaling.

Supporting this idea, tizanidine, an antispastic that does not activate the GABA-B receptor, failed to induce any significant change in MSC differentiation. The lack of effect of tizanidine on adipogenic and osteochondrogenic gene expression may be attributed to the signaling characteristics of its primary target, the α2-adrenergic receptor [50]. Consistently, tizanidine did not significantly alter lineage-associated markers relative to controls—except for *COLX*—suggesting that α2-adrenergic signaling does not drive transcriptional programs underlying MSC commitment. In line with these findings, protein-level analysis of SPP1 revealed no differences between control and tizanidine-treated cells, indicating that tizanidine does not modulate SPP1 expression, likely due to its inability to activate GABA-B receptor-mediated pathways. This aligns with the current literature, which does not support a direct role for tizanidine in these differentiation pathways.

The isolated modulation of *COLX* likely reflects a modest or context-dependent effect rather than the induction of a coordinated differentiation response. Taken together, these observations suggest that, under the conditions tested, tizanidine is unlikely to act as a major regulator of adipogenic or osteochondrogenic differentiation in MSC C3H10T1/2 cells and might be less likely to contribute to processes associated with HO, being potentially safer to manage spasticity.

To evaluate whether baclofen can modulate MSC differentiation even under conditions that strongly oppose osteoblastogenesis, C3H10T1/2 cells were also exposed to strong inflammatory stimuli. Both IL-1β and LPS increased the expression of adipogenic markers, while the expression of osteochondrogenic genes were only partially decreased. This result reflects the harmful effect of elevated inflammatory signals, which create an environment that disrupts normal endochondral differentiation and inhibits osteoblast differentiation [23,25,51]. However, the magnitude of these effects differed between stimuli, with LPS inducing a higher adipogenic gene expression. In contrast, IL-1β promoted a stronger reduction in the expression of mid-to-late osteochondrogenic markers such as *COLX* and *BGLAP* [9,52,53], while early-stage markers such as *COL2A1* and *SOX9* [9,54] were barely affected.

These differences might be related to the origin of these stimuli. LPS represents an exogenous pathogen-associated molecular pattern (PAMP), and its detection requires a complex extracellular recognition system [55], whereas IL-1β is an endogenous cytokine, activating the innate immune response through a different mechanism [56]. Regardless of the nature of the stimulus applied, baclofen co-administration consistently reduced the induction of adipogenic markers, which supports the idea that even under strongly antagonistic conditions, baclofen is able to induce some kind of cellular reprogramming and shift MSC differentiation away from adipogenesis, promoting the differentiation towards alternative lineages. To note, inflammation in the presence of baclofen is not capable of significantly altering osteochondrogenesis. Although baclofen did not significantly induce osteochondrogenic genes or SPP1, it appeared to attenuate the reduction of these markers caused by inflammatory stimuli, suggesting a stabilizing effect on osteochondrogenic programs.

While our findings provide new insight into the effects of baclofen on MSC fate, several limitations must be acknowledged. The study was conducted in vitro using a single murine MSC cell line, which does not fully recapitulate the complexity of the in vivo environment. Key contributors to HO development—such as biomechanical stimuli, vascularization, and systemic regulatory signals—are not represented in this model. Consequently, the results should be interpreted within the constraints of a reductionist experimental system. Moreover, although HO marker analysis was included, it was limited to a single marker and does not encompass the full spectrum of molecular changes associated with MSC differentiation.

Furthermore, while consistent changes were observed in adipogenic and osteochondrogenic gene expression, as well as in SPP1 secretion, alternative interpretations should be considered. The observed shift in lineage commitment may not be exclusively attributable to direct GABA-B receptor activation but could also involve secondary signaling pathways or context-dependent modulation of MSC plasticity, particularly under adipogenic or inflammatory conditions. Detailed analysis of downstream intracellular signaling cascades will be necessary to define the molecular mechanisms underlying these effects.

Although previous reports support a role for GABA-B signaling in skeletal tissues, its function appears to be context-specific and remains incompletely characterized. Variability in cellular models, differentiation stage, and experimental conditions may account for differences across studies. Therefore, our findings should be interpreted within the framework of the present model. From a translational perspective, the present data do not establish a causal relationship between baclofen administration and HO development in patients. Rather, they indicate that pharmacological modulation of GABA-B signaling can influence MSC lineage decisions under defined experimental conditions. Confirmation in human MSCs and in vivo models will be necessary to determine the true biological and clinical implications of these findings.

Within these limitations, our results provide evidence that baclofen can directly modulate MSC fate independently of spasticity, by shifting differentiation away from adipogenesis and inducing a cellular reprogramming that might be relevant for regulating ectopic bone formation during the early stages of HO. On the other hand, tizanidine showed minimal effects on gene expression. Therefore, these findings highlight a potential clinical risk associated with baclofen use and provides new knowledge to be considered when selecting which drug to use to manage spasticity in patients at risk of HO, indicating tizanidine’s potential advantage as an antispastic in clinical settings, where minimizing the risk of ectopic bone formation is critical.

## 4. Materials and Methods

### 4.1. Reactives

All cell culture reagents and treatments, unless otherwise specified, were purchased from Sigma-Aldrich (St. Louis, MO, USA), including high-glucose Dulbecco’s Modified Eagle Medium (DMEM), penicillin/streptomycin, L-glutamine, fetal bovine serum (FBS, lot BCBW6329), trypsin, baclofen and tizanidine.

The inflammatory agents used in this study were LPS from Escherichia coli O26:B6, also purchased from Sigma-Aldrich, and IL-1β, obtained from Abcam (Cambridge, UK). The mouse mesenchymal stem cell (MSC) line C3H10T1/2 was gently provided by Dr. Pardo (IDIS Institute, Santiago de Compostela, Spain).

### 4.2. Cell Culture

For the development of this study, the MSC cell line C3H10T1/2 was used, selected for its proven ability to differentiate into both the osteoblastic and adipocytic lineages [21].

These cells were cultured in high-glucose DMEM (Dulbecco’s Modified Eagle’s Medium) supplemented with 10% fetal bovine serum (FBS), 4 mM L-glutamine, and 100 U/mL penicillin/streptomycin. They were maintained in the culture medium on adherent plates (Corning, Glendale, AZ, USA) in a controlled atmosphere at 37 °C with 5% CO_2_ under conditions of saturated humidity.

### 4.3. Differentiation Studies and Treatments

For all assays, cells were seeded in 24-well plates at a density of 10^4^ cells per well. The differentiation was initiated 6 h after seeding, and the treatments were added simultaneously.

Adipogenic differentiation condition was established following the protocol optimized by Alonso-Pérez et al. (2022) [21], which describes the use of the C3H10T1/2 cell line as a model to study the adipogenic–osteoblastic balance. Adipogenesis was acquired after 7 days (Figure S1).

Baclofen (100 μM) or Tizanidine (10 μM) were added during the adipogenesis. These concentrations were selected to maximize receptor activation and ensure a detectable functional effect on the cells, based on previous in vitro studies [57,58]. Gene expression was compared to an untreated control. Based on the results obtained, the ability of baclofen to modulate the gene expression profile of C3H10T1/2 adipogenesis was evaluated under different pathophysiological contexts: basal and inflammatory conditions. Inflammation was induced using two different agents: IL-1β (0.5 ng/mL) or LPS (100 ng/mL).

### 4.4. Gene Expression Analysis

For RNA extraction, cells were lysed using Tri-Reagent (Sigma-Aldrich, St. Louis, MO, USA), and RNA was purified with the EZNA Total RNA Kit I (Omega, Bio-Tek, Inc., Norcross, GA, USA), following the manufacturer’s instructions. RNA samples were treated with DNase I (Lucigen, Middleton, WI, USA) to eliminate potential genomic DNA contamination. Only samples with a 260/280 nm ratio greater than 2 and a 260/230 nm ratio greater than 1.7 were used in gene expression analyses to ensure a suitable purity.

Complementary DNA (cDNA) synthesis was performed from 50 ng of RNA using the High-Capacity cDNA Reverse Transcription Kit (Applied Biosystems, Life Technologies, Grand Island, NY, USA), following the manufacturer’s instructions.

The cellular response to treatments was evaluated by measuring the relative mRNA expression levels of 9 marker genes using quantitative real-time polymerase chain reaction (RT-qPCR). All experiments were performed using three independent biological replicates (n = 3), where each replicate represents an independent differentiation and treatment of C3H10T1/2 cells.

The panel of genes studied included key markers for differentiation into both adipocyte and chondroosteoblast lineages. The adipogenic marker genes selected were FABP4, PLIN-2, ADIPOQ and PPARG (Table 1). The osteochondrogenic marker genes studied were COL2A1, SOX9, COLX, BGLAP and PTHR (Table 1).

Amplification was performed using Bio-Rad (Hercules, CA, USA) MasterMix and the primers (Sigma-Aldrich, St. Louis, MO, USA) shown in Table 1. Data analysis was conducted with QuantStudio™ Design and Analysis Software v1.6.1 (Applied Biosystems, Life Technologies, Grand Island, NY, USA). Relative quantification was obtained using the comparative ∆∆Ct method, with hypoxanthine-guanine phosphoribosyl transferase 1 (HPRT) serving as the reference gene for normalization. It was confirmed that HPRT expression was not modulated throughout the experimental procedures in the cell lines used.

### 4.5. Protein Expression Analysis

Once the corresponding incubation time was completed, the supernatant was collected and processed for albumin depletion by treatment with dithiothreitol (DTT) (Bio-Rad, Hercules, CA, USA), achieving a final concentration of 500 mM. After incubation for 1 h at room temperature and centrifugation at 14,000× *g*, the supernatant was recovered, and proteins present in it were subsequently precipitated. For this purpose, 200 µL of supernatant were mixed with 800 µL of 100% methanol (Sigma-Aldrich, St. Louis, MO, USA), 200 µL of chloroform (Sigma-Aldrich, St. Louis, MO, USA), and 600 µL of Milli-Q water. The mixture was vortexed to homogenize the solvents and subsequently centrifuged at 10,000× *g* for 5 min to allow phase separation.

After centrifugation, the upper phase was discarded and 600 µL of 100% methanol (Sigma-Aldrich, St. Louis, MO, USA) were added. The mixture was vortexed again to disrupt the interphase membrane and ensure proper mixing of the solvents. It was then centrifuged again at 10,000× *g* for 10 min to induce protein precipitation.

Finally, the supernatant was discarded, and the protein pellet was resuspended in 50 µL of RIPA buffer (Merck, Darmstadt, Germany), supplemented with protease and phosphatase inhibitors (Thermo Fisher Scientific, Waltham, MA, USA), in order to prevent protein degradation and preserve the phosphorylation state during extraction.

Protein analysis was performed using the automated Jess system (ProteinSimple, Bio-Techne, MN, USA), which allows protein detection and quantification by capillary electrophoresis (Simple Western). Samples were mixed with a fluorescent master mix containing DTT, a size marker, and loading buffer.

The mixtures were heated at 95 °C for 5 min to denature the proteins. Subsequently, the plate was loaded with the samples along with primary and secondary antibodies, detection reagents, and buffer solutions, following the manufacturer’s instructions

The primary antibody used was rabbit anti-SPP1 (Cell Signaling Technology, Danvers, MA, USA). The assay was carried out following the standard protocol using Compass for Simple Western software v7.1.0.0 (ProteinSimple, Bio-Techne, Minneapolis, MN, USA), which was also used for data analysis. All protein expression experiments were performed using three independent biological replicates (n = 3), corresponding to independent differentiations and treatments of C3H10T1/2 cells. Protein quantification was performed by calculating the area under the curve (AUC) of each signal peak, with values normalized to the control.

### 4.6. Fluorescence Microscopy Analysis

After the designated incubation period, the supernatant was carefully removed, and the cells were fixed with 3–4% formaldehyde (Thermo Fisher Scientific, Waltham, MA, USA) for 10 min at room temperature, followed by washes with Phosphate-Buffered Saline (PBS) (Thermo Fisher Scientific, Waltham, MA, USA). Subsequently, NucBlue™ Live ReadyProbes™ (Hoechst 33342) (Invitrogen, Carlsbad, CA, USA) was added at a concentration of 1 drop per mL of PBS (according to the manufacturer’s protocol), which corresponds approximately to 21 μL per mL of PBS, and incubated for 3 min to stain cell nuclei blue, followed by additional PBS washes. Finally, LipidTOX (Invitrogen, Carlsbad, CA, USA) was applied to stain lipid droplets red, and the cells were incubated at room temperature for 30 min before imaging, following the manufacturer’s instructions. Images were captured using the Cytation 5 imaging system (Agilent Technologies, Santa Clara, CA, USA).

### 4.7. Statistical Analysis

Gene expression levels were normalized to untreated control. Data is presented as mean ± standard error of the mean (SEM) from at least three independent replicates. Statistical analyses were performed using GraphPad PRISM 8 software, version 8.0.2 (GraphPad Software Inc., La Jolla, CA, USA). Statistically significant differences between experimental conditions were assessed using Student’s *t*-test, with *p*-values < 0.05 considered significant.

## Figures and Tables

**Table 1:** Primers used for RT-qPCR.

Description	Symbol	Forward Primer (5′-3′) Sequence	Reverse Primer (5′-3′) Sequence
**Mouse Fatty acid binding protein 4**	*FABP4*	GTAAATGGGGATTTGGTCAC	TATGATGCTCTTCACCTTCC
**Mouse Fatty acid binding protein 4**	*PLIN*-2	ATAAGCTCTATGTCTCGTGG	GCCTGATCTTGAATGTTCTG
**Mouse Adiponectin**	*ADIPOQ*	CCACTTTCTCCTCATTTCTG	CTAGCTCTTCAGTTGTAGTAAC
**Mouse Peroxisome proliferator activator receptor γ**	*PPARG*	AAAGACAACGGACAAATCAC	GGGATATTTTTGGCATACTCTG
**Mouse Collagen Type II Alpha 1 Chain**	*COL2A1*	GCGATGACATTATCTGTGAAG	TATCTCTGATATCTCCAGGTTC
**Mouse SRY-Box Transcription Factor 9**	*SOX9*	CTCATTACCATTTTGAGGGG	AAAATACTCTGGTTGCAAGG
**Mouse Collagen Type X Alpha 1 Chain**	*COLX*	TCATGGGATGTTTTATGCTG	TCTTACTGGAATCCCTTTACTC
**Mouse Bone Gamma-Carboxyglutamate Protein**	*BGLAP*	ACCATGAGGACCATCTTTC	GGACATGAAGGCTTTGTC
**Mouse Parathyroid Hormone Receptor**	*PTHR*	AGAGAAGAAGTATCTGTGGG	GATAAAGAGGATGAAGTTGAGC
**Mouse Hypoxanthine Phosphoribosyl transferase 1**	*HPRT*	AGGGATTTGAATCACGTTTG	TTTACTGGCAACATCAACAG

## Data Availability

The original contributions presented in this study are included in the article/Supplementary Materials. Further inquiries can be directed to the corresponding author.

## Supplementary Materials

The following supporting information can be downloaded at: https://www.mdpi.com/article/10.3390/ijms27062783/s1.

## Abbreviations

The following abbreviations are used in this manuscript:

*ADIPOQ*	Adiponectin
AUC	Area under the curve
*BGLAP*	Bone gamma-carboxyglutamate protein
CNS	Central nervous system
*COL2A1*	Collagen type II alpha 1
*COLX*	Collagen type X
DMEM	Dulbecco’s Modified Eagle Medium
DTT	Dithiothreitol
*FABP4*	Fatty Acid Binding Protein 4
FBS	Fetal bovine serum
FOP	Fibrodysplasia ossificans progressiva
GABA	γ-aminobutyric acid
HO	Heterotopic Ossification
*HPRT*	Hypoxanthine-guanine phosphoribosyl transferase 1
IL-1β	Interleukin-1β
LPS	Lipopolysaccharides
MSC	Mesenchymal cell
PAMP	Pathogen-associated molecular pattern
*PLIN2*	Perilipin-2
POH	Progressive osseous heteroplasia
*PPARG*	Peroxisome Proliferator-Activated Receptor Gamma
*PTHR*	Parathyroid hormone 1 receptor
RT-qPCR	Quantitative real-time polymerase chain reaction
SCI	Spinal cord injury
SEM	Standard error of the mean
*SOX9*	SRY-box transcription factor 9
SPP1	Secreted Phosphoprotein 1/Osteopontin
