# Supplementary material for: Baclofen Promotes Osteochondrogenic Commitment of Mesenchymal Stem Cells: Implications for Heterotopic Ossification Risk
Source: Int J Mol Sci. 2026 Mar 19;27(6):2783. doi: 10.3390/ijms27062783 (PMC13027275; doi:10.3390/ijms27062783)
Supplement: Supplementary file 1 [file ijms-27-02783-s001.zip › ijms-4174933-supplementary.pdf]

Supplementary figure 1

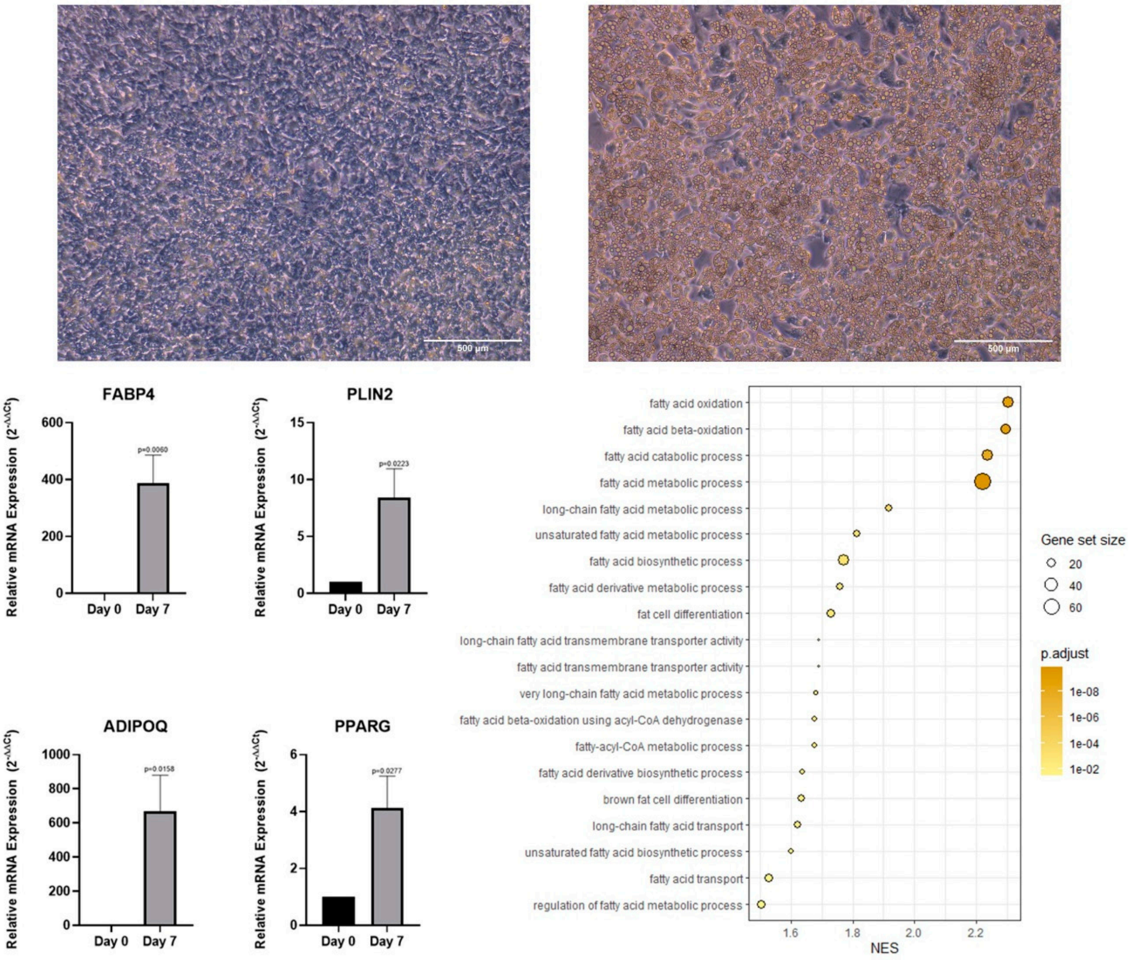

Figure S1. Validation of adipogenic differentiation in C3H10T1/2 cells. (A) C3H10T1/2 cells at day 0 and C3H10T1/2 cells after 7 days of adipogenic differentiation. No staining or treatment was applied. **(B)** Relative mRNA expression of canonical adipogenic markers in undifferentiated cells (Day 0) and after 7 days of adipogenic differentiation. **(C)** Pathway analysis of SWATH–MS proteomics results. Proteomic changes induced in C3H10T1/2 cells during a 7-day adipogenic differentiation compared to other samples, analysed using KEGG and Reactome databases.
